# Ecological Considerations When Designing Mitigation Translocations: An Australian Reptile Case Study

**DOI:** 10.3390/ani13162594

**Published:** 2023-08-11

**Authors:** Holly S. Bradley, Michael D. Craig, Sean Tomlinson, Adam T. Cross, Michael J. Bamford, Philip W. Bateman

**Affiliations:** 1 ARC Centre for Mine Site Restoration, School of Molecular and Life Sciences, Curtin University, Kent Street, Bentley, Perth, WA 6102, Australia; 2 School of Biological Sciences, University of Western Australia, Crawley, WA 6009, Australiabamford.consulting@iinet.net.au (M.J.B.); 3 School of Environmental and Conservation Sciences, Murdoch University, Perth, WA 6150, Australia; 4 School of Molecular and Life Sciences, Curtin University, Kent Street, Bentley, Perth, WA 6102, Australiaadam.cross@curtin.edu.au (A.T.C.); 5 School of Biological Sciences, University of Adelaide, North Terrace, Adelaide, SA 5000, Australia; 6 Ecological Health Network, 1330 Beacon St, Suite 355a, Brookline, MA 02446, USA; 7 Bamford Consulting Ecologists, 23 Plover Way, Kingsley, WA 6026, Australia; 8 Behavioural Ecology Laboratory, School of Molecular and Life Sciences, Curtin University, Kent Street, Bentley, Perth, WA 6102, Australia

**Keywords:** mitigation translocation, reptile, ecology, habitat

## Abstract

**Simple Summary:**

A common method of alleviating impending threats to wildlife populations is to relocate them from danger, which is known as mitigation translocation. However, these translocations have high failure rates because they lack appropriate funding, resources, and a knowledge of species requirements. Here, we use the endangered western spiny-tailed skink (*Egernia stokesii badia*) as a case study to exemplify how targeted ecological research can be used to help inform translocation planning. We found that the skinks have specific requirements for predator management, foraging and prey availability, and log pile structures, which can all help improve the targeted selection of translocation sites in the future. Application of a similar scientific framework to planning is likely to improve mitigation translocation success for a range of threatened species.

**Abstract:**

Translocation science has made considerable progress over the last two decades; however, reptile translocations still frequently fail around the world. Major knowledge gaps surround the basic ecology of reptile species, including basic factors such as habitat preference, which have a critical influence on translocation success. The western spiny-tailed skink (*Egernia stokesii badia*) is used here as a case study to exemplify how empirical research can directly inform on-ground management and future translocation planning. A combination of studies, including LiDAR scanning of microhabitat structures, camera trapping, plasticine replica model experiments and unbounded point count surveys to assess predation risk, and visual and DNA analysis of dietary requirements, were all used to better understand the ecological requirements of *E. s. badia*. We found that the skinks have specific log pile requirements, both native and non-native predator management requirements, and a largely herbivorous, broad diet, which all influence translocation site selection and management planning. The use of *E. s. badia* as an Australian case study provides a clear strategic framework for the targeted research of meaningful ecological factors that influence translocation decision-making. Similar approaches applied to other reptile species are likely to fundamentally increase the capacity for effective management, and the likelihood of future successful translocations.

## 1. Introduction

Translocation biology is a growing field [1,2] and is a major conservation tool used to help safeguard threatened species [3,4]. A marked increase in the number of translocation publications since the year 2000, however, has not corresponded with an advancement in translocation practices [1,5,6]. This has been attributed to the largely ad hoc nature of translocation methods [1], which could be improved by integrating translocation projects within more holistic scientific frameworks of conservation biology [7].

Herpetofauna have often been overlooked by translocation reviews [8], with research biased towards mammals and birds [9]. There has been debate within the scientific community on the suitability of reptiles for translocation, which appear to have low success rates compared with other taxa [8,10,11,12]. Over the past thirty years, however, there has been a two-fold increase in the number of published accounts of successful amphibian and reptile translocations [8], indicating reintroduction biology to be just as viable a conservation tool for reptiles as for other taxonomic groups [13,14]. Despite this positive trend, there are still significant knowledge gaps that can impede translocation success [14,15,16]. Of all reptile species assessed using IUCN Red List Criteria (4648 species as of 2016), 19% are recorded as Data Deficient [15]. There is a particular lack of data surrounding movement and habitat requirements, which are two of the most significant factors contributing to reptile translocation failure [8,14].

The knowledge gaps surrounding reptiles are particularly evident in Australia because reptile conservation research historically has concentrated in the northern hemisphere [16,17]. Despite Australia being a global hotspot for reptile species richness, harbouring approximately 10% of all currently described reptile species globally and more species than any other country in the world [18,19,20], there remains a critical lack of knowledge about Australian reptile biology and ecology. Much of Australia’s high reptile diversity is concentrated in remote, sparsely populated arid and semi-arid ecosystems [20,21], and ecological research on terrestrial reptiles is largely constrained by the accessibility of habitat [22]. Hence, reptile species are under-represented in recovery planning in Australia [23], which may deprive them of conservation investment as species with recognised conservation caveats or threat listings are more likely to receive management and investment than non-threatened or unassessed species [24]. Therefore, despite high richness, reptiles are under-represented in conservation research and planning, both in Australia and globally [15,18].

The Australian population is highly urbanised [25], which places substantial conservation pressure on some Australian ecosystems [26]. Even in the less populated arid and semi-arid areas, there are pressures other than urban growth on biodiversity, including agriculture, pastoralism and mining operations, such as in Western Australia which harbours significant reptile richness [27,28,29]. Wildlife relocations are often promoted as a mitigation tool to prevent mortality from sites to be cleared for urbanisation or mineral extraction [27,30,31,32]. However, most mitigation translocations are unsuccessful, failing to establish self-sustaining populations [33,34,35] due to a lack of detailed knowledge on the establishment, persistence, metapopulation and ecosystem-level requirements of a species prior to the translocation [27,36]. This is because mitigation translocations are often proposed by development agencies, constrained to timeframes consistent with the development schedule, and are often naïve to the pace of ecological processes and the realistic timeframes that this imposes on success. Without an informed ecological knowledge base to support planning, even well-intentioned mitigation translocation attempts risk undermining biodiversity conservation efforts [33]. Worse, without an appropriate understanding of ecological processes, monitoring efforts to assess translocation success, if they are deployed at all, may not be scheduled appropriately, resulting in a lack of reporting of success and failures [3]. Yet, as we have established, the knowledge gaps underpinning reptile translocation are substantial, and research priorities and approaches to address these remain open to interpretation.

Here, we integrate the findings of a three-year research program on the western spiny-tailed skink (*Egernia stokesii badia*) as a case study of undertaking a detailed, scientific approach to identify and address knowledge gaps on the ecological requirements of a threatened reptile in Australia to help inform future translocations. This research program has provided empirical evidence on the ecology of the skink, including the predators of *E. s. badia*, particularly those which target the log piles used as refuges by the skinks, and how predator activity is influenced by proximity to human-generated waste landfill. We also consider the generation of detailed knowledge of the microhabitat requirements of the skinks, allowing us to identify optimal release sites through the novel use of LiDAR as a measurement tool to quantify structural features that promote occupancy by skink colonies. Finally, we consider the plant and invertebrate diet of the skink and evidence for an ontogenetic dietary shift with subadult skinks appearing to target high-reward prey items, and adult skinks having a more opportunistic supplementation of their herbivorous diet with invertebrates, through visual identification and DNA metabarcoding. We then discuss the management implications of these findings, particularly relating to translocation.

## 2. Species Ecology

**Figure 1:**
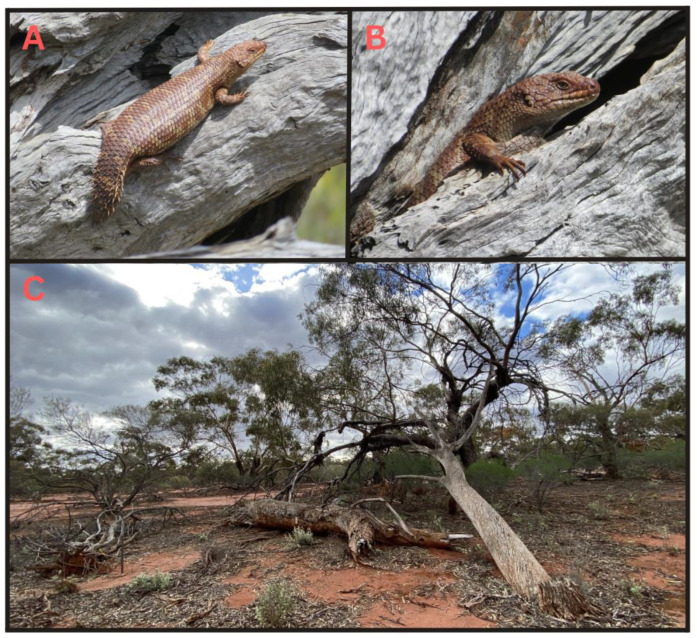
Image examples of *Egernia stokesii badia* (**A**,**B**) and occupied log pile habitat (**C**) in the semi-arid Mid West region of Western Australia. Photos taken by H.S. Bradley.

*Egernia stokesii badia* is an endangered subspecies of skink, endemic to the arid and semi-arid regions of Western Australia [37]. The skinks can live in family groups in log piles [37,38] (Figure 1). Beyond this basic information, most ecological knowledge of the subspecies is largely inferred from what is known of other *Egernia stokesii* subspecies, despite *E. s. badia* having a different distribution and living in naturally occurring piles of fallen logs rather than rock crevices [27,37].

As a large part of the range of *Egernia stokesii badia* is in the Mid West region of Western Australia, much of which is covered by active or prospective mining tenure, it is likely that future translocation of some populations will occur due to clearing for mineral exploration and extraction activities [37], though, to our knowledge, there are no records of successful translocations of this subspecies. Broader requirements, such as the provision of a similar habitat and of a log pile for translocated colonies is understood; however, further investigation into the specific ecological requirements of *E. s. badia* is required to understand why previous translocations may have been unsuccessful, and how to improve translocation methodology for the future. For this reason, this study focused on determining the optimal log pile characteristics, diet requirements, and what predators of the skinks are likely to require targeted management, to help optimise future translocation efforts and avoid detrimental impacts to skink abundance and distributional extent.

### 2.1. Predation as a Translocation Risk

Globally, predation by native predators is a major cause of translocation failure [39,40,41]. Introduced predators are also a major cause of and contributing factor towards translocation failures in Australia, e.g., [42,43,44]. For this reason, many Australian translocation sites are on offshore islands [45,46,47] or in predator-proof enclosures, e.g., [46,47,48,49] to reduce the risk of translocation failure. Other Australian trials have implemented targeted control of invasive species to reduce the likelihood of high mortalities, e.g., [50,51]. However, to understand the appropriate management to implement, and to target management according to specific predatory species, it is important to first understand what predators present the greatest threat to the focal species. For this study, a combined experimental approach was employed to determine both the types of predators and behaviour of predators, according to habitat and anthropogenic infrastructure.

The influence of the presence of log piles on predator behaviour towards *E. s. badia* was assessed through plasticine model experiments, unbounded point count bird surveys and camera trapping [52]. An equal number of sites with and without log piles were selected, and replica plasticine models of *E. s. badia* were placed at each site type to assess predation pressure in relation to log piles. Unbounded point count bird surveys were also conducted at sites with and without log piles, and sites with log piles inhabited by *E. s. badia*, to determine the relative activity of predatory bird abundance according to site type. Camera traps were placed at the three site types to capture as broad a range of predators as possible, to assess relative activity, and determine predator behaviour at different site types [52].

The major findings for predation management relating to *E. s. badia* translocation pertained to the following: (i) translocation site selection; and (ii) the targeted management of predators [27]. Firstly, two predators of particular threat that we identified for the skink were feral cats (*Felis catus*) [37,53,54] and corvids (*Corvus orru*, *C. bennetti*, and *C. coronoides*) [27]. Feral cats are a widely recognised threat to biodiversity [55,56] and to translocation success [42,44,57] in many regions, but corvid control is rarely considered in translocation proposals. Globally, native corvid populations can become overabundant in anthropogenically modified landscapes with increased food resources, such as at landfill sites [27,58,59,60]. This was also evident in this study, where the relative activity of corvids increased with proximity to landfill [52]. Methods to avoid overabundance of native corvids would, therefore, be valuable, perhaps through the management of anthropogenic food sources. Nevertheless, translocation sites for skink colonies evidently need to be as distant as possible from potential anthropogenic food sources, such as landfill sites, with their role in supplementing and augmenting populations of generalist predators such as corvids and feral cats [61,62]. Translocation site selection away from linear infrastructure such as powerlines, which are correlated with an increase in corvid abundance in the U.S.A. [63,64], is also likely to be important in reducing potential predation pressure, as individuals are more vulnerable to predation post-translocation [27].

Targeted management to control the abundance of predators in areas identified for translocation is also likely to be necessary. For example, targeted lethal control of invasive feral cats, through baiting [65,66], shooting [67,68], or trapping [67,69], near translocation areas could help to reduce predation risk for translocated skinks [27]. Feral cats were shown to be capable of killing adult and subadult skinks [52] and were also one of the predators with the greatest relative activity seen at all site types. Both corvids and feral cats are attracted to novel objects and areas [70,71,72,73], such as translocation sites. Controlling overabundant and invasive predators would, therefore, be a priority, as any site modifications to enhance the structure of log piles may attract these predators and increase the risk of translocation failure [27].

### 2.2. Microhabitat Requirements in Identifying or Constructing Recipient Locations

To determine the structural microhabitat requirements of *E. s. badia*, three-dimensional laser scans of uninhabited and inhabited log piles were taken. The laser scanning data were collected using a terrestrial LiDAR scanner, the Maptek^TM^ I-Site^TM^ 8800 (Maptek, Adelaide, Australia), placed in three to five positions around each log pile, depending on how large the log pile was [74]. The overlapping scans were then merged into a single point cloud for analysis to create a full 360-degree view of target log piles. The (i) maximum canopy height, (ii) number of logs, (iii) length of log system, (iv) number of branches above and below/adjacent to the main log, (v) log structure height, (vi) diameter of widest hollow, (vii) presence of overhanging vegetation, (viii) position of the log pile (majority resting on ground or raised), (ix) orientation of the log pile, and (x) diameter of the widest section of log were then compared using multiple logistic regression models to determine trends in the types of log piles skinks selected [74].

**Figure 2:**
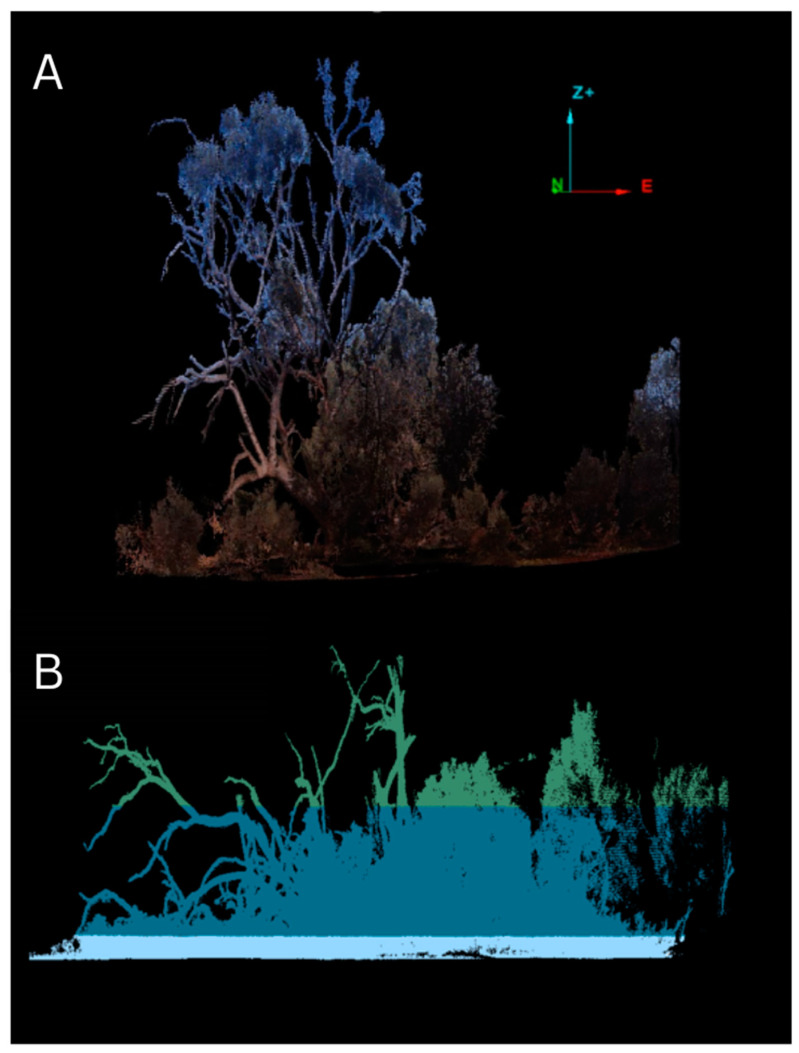
Example of (**A**) a side-view of a three-dimensional point cloud with photographic colouring generated from scanning a log pile site; and (**B**) a side view of a separate point cloud scan, where the layers have been divided into ground cover, mid-storey, and canopy layers.

LiDAR analysis indicated that *Egernia s. badia* has specific microhabitat requirements that must be considered when selecting or modifying an optimal translocation site. Skink occupancy was highest in longer log piles with an average of two logs with some overhanging vegetation, preferably at a mid-storey height and reduced at canopy height [74] (Figure 2). This is likely to ensure enough space for segregation between members of the skink colony, which vary in size between juveniles, adults and gravid females [75]. Overhanging vegetation is also likely to be important for providing microhabitat variability, thermal buffering and temperature gradients to support behavioural thermoregulation [74,76]. This is important in an arid landscape with scattered vegetation [74,76], where daytime temperatures can often exceed the upper thermal tolerance limits of most reptiles [74,77].

In some cases, canopy cover can provide perches for ambush predators [74,78,79]. As corvids increased in relative activity and focused their hunting behaviour around log piles inhabited by *E. s. badia*, this potentially indicates that the trend for log pile selection with reduced canopy cover could be associated with fewer perching options for avian predators [74]. Overall, when selecting or modifying translocation sites, microhabitat characteristics that support optimal thermoregulation, predator refuge and social segregation are potential considerations to help maximise the likelihood of establishment and persistence [74].

### 2.3. Dietary Requirements and Translocation Site Selection

*Egernia stokesii badia* was predicted to have an opportunistic and highly varied diet. Ontogenetic resource partitioning was also expected to allow for the coexistence of juveniles with adults in a permanent log pile shelter. We tested these predictions using DNA metabarcoding and visual examination of scats. A total of 30 scats (14 adult and 16 subadult) were collected from five active colonies in August 2018 for visual dissection [27]. Each of these scats was placed in a Petri dish with water, and forceps were used to gently tease the contents apart. Each scat was then separated into invertebrate material and other (mostly plant) material, using a dissecting microscope [27]. The material was then air dried for 48 h and the dry weights were recorded. For the visual identification component, the invertebrate taxa were identified to the level of order [27].

For the genetic analysis, 36 scats (18 adult and 18 subadult) were collected from nine different colonies in September 2019 [27]. DNA was extracted from each scat using a Qiagen PowerFecal Pro kit (Qiagen). Fusion tag primers were used to develop sequencing libraries and the bioinformatic pipeline eDNAFlow [80] was used to analyse raw sequence data generated from the metabarcoding. Where a species-level taxonomic assignment was made, the sequence similarity was checked [27]. When the match was <97%, the assignment was dropped back to the genus level. Taxonomic nomenclature was validated using the Global Biodiversity Information Facility [81], with the final taxa list converted into a presence/absence matrix for each assay [27].

**Figure 3:**
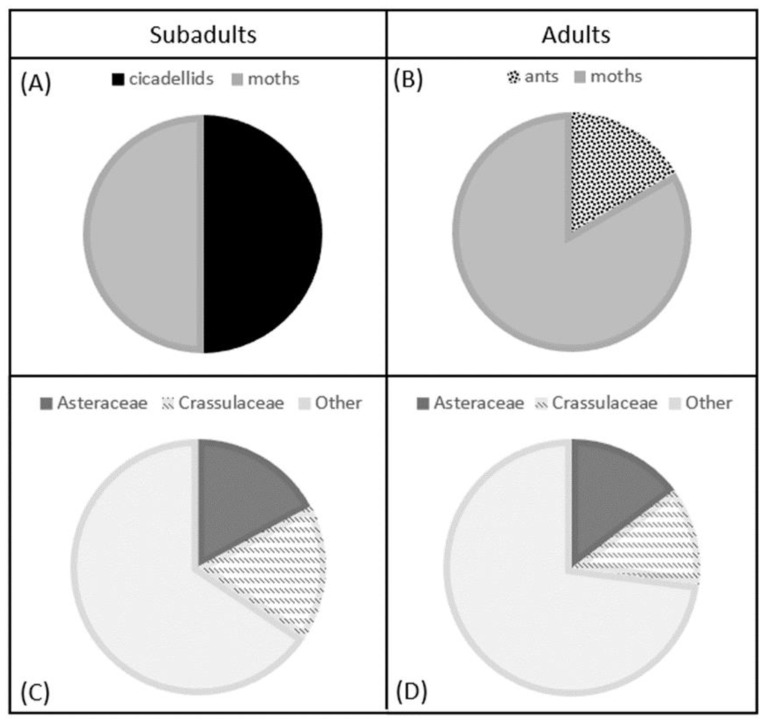
Pie charts as taken from [27], highlighting the proportion of invertebrate groups in subadult (**A**) and adult (**B**) *Egernia stokesii badia* scat samples, and the most abundant plant families in subadult (**C**) and adult (**D**) scat samples, as identified through DNA metabarcoding.

*Egernia stokesii badia* is predominantly herbivorous (approximately 91%), supplemented by the opportunistic consumption of invertebrates, except in the case of subadults, which appeared to directly target some invertebrate prey items of high nutritional value (e.g., Cicadellidae) possibly to facilitate rapid growth and development (Figure 3) [27]. While the skinks fed from a high diversity of food plants, they particularly favoured the Asteraceae, both as adults and subadults [27]. Many Asteraceae are small, soft, annual, flowering plants (e.g., *Isoetopsis graminifolia*) [82], and can be highly abundant in the spring months in Western Australia [83]. Their high abundance in the diet probably reflects their high abundance in the landscape during this time. Another highly abundant plant family in the skink diet was Crassulaceae, generally characterised by plants with fleshy, succulent leaves [82]. In contrast to Asteraceae, Crassulaceae have a narrower niche breadth, often occurring in moist, shaded areas [84,85], and were, therefore, probably specific foraging targets of the skinks. The Crassulaceae likely offers a source of both nutrients and water, which has been suggested as valuable for other reptiles persisting in arid habitats [27,86].

Provision of a high floristic diversity including annual species, plus microhabitat complexity to support the growth of more specialist plants such as *Crassula* spp., is likely critical to support the foraging requirements of an *E. s. badia* colony [27]. A healthy ecosystem which supports a diverse invertebrate community is also likely to be an important consideration for translocation site selection, particularly to support the growth and development of younger subadults [27].

## 3. Significance of the Research Program

**Figure 4:**
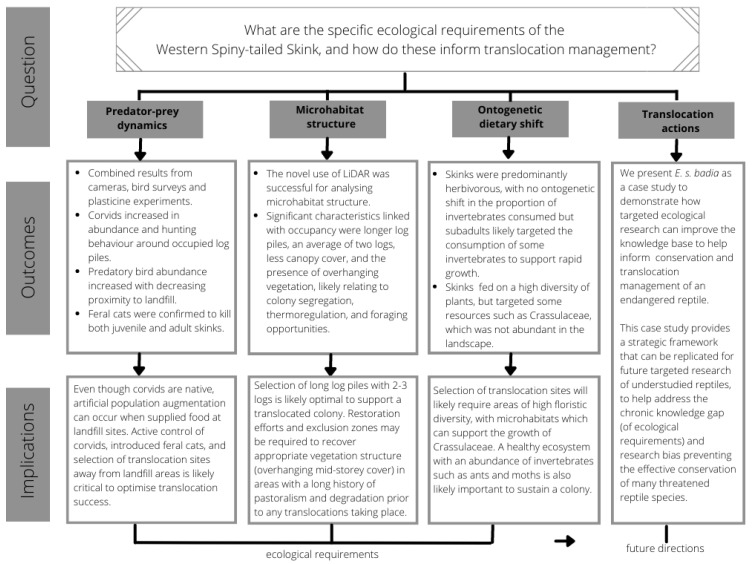
Conceptual framework highlighting the overall research question, ecological requirements investigated, summarised outcomes, and wider implications of this case study. Modified from [27].

The common assumption underpinning translocation and restoration biology is that the return of floristic diversity, and provision of basic habitat structures, such as vegetation and logs, will lead to the return of fauna on their own [87]. However, research has found that whilst this may be a useful first step in the recreation, or selection, of a translocation site or area for recolonisation, this hypothesis on its own can be unreliable due to the complexity of faunal ecological requirements [88,89,90,91]. As such, a more specific understanding of key limiting factors to the successful establishment of many fauna species at a release site or restored area is vital. In this project, new knowledge on the log pile and vegetation structural requirements of a translocation site, food plants required in proximity to the refuge site, and the need for targeted predator management at a selected translocation site have been determined. This significantly improves basing translocation site selection through a broad understanding of suitable vegetation type [92]. Overall, this case study provides a strategic framework and an example of a novel application of technology that can be replicated for future targeted research of understudied species to help address the chronic knowledge gap and research bias preventing the effective conservation of many threatened reptile species (Figure 4) [27].

**Figure 5:**
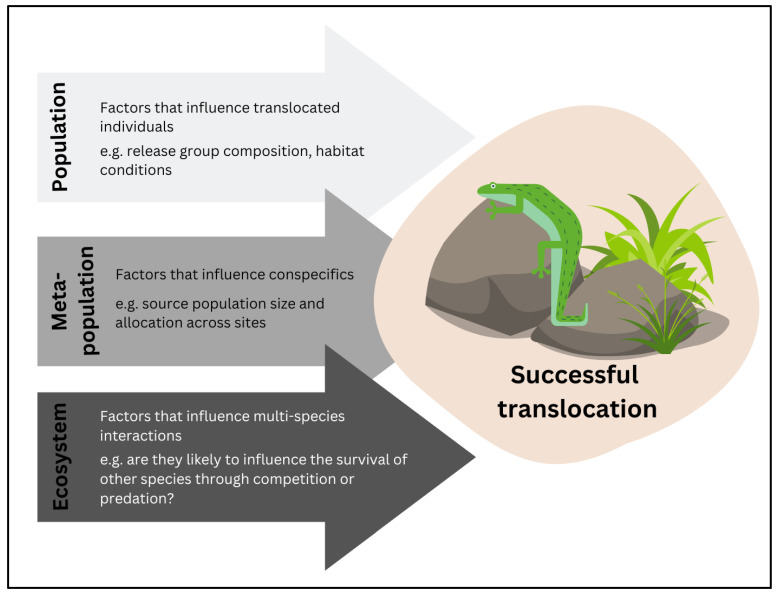
Summary of key research considerations in translocation biology, as adapted from Armstrong and Seddon [36]. Developed from images created using © Procrea, © sketchify, © OpenClipart-Vectors and © imaginaryparty via Canva.com.

The research program that we summarise here is among the first to follow the call for mitigation translocations to follow the same scientific rigour and framework as is expected for conservation translocations [33,36]. Armstrong and Seddon [36] appealed for translocation biologists to consider the biological requirements of the target species when understanding ‘habitat’, and not just focus on the easier, rapidly assessable landscape features such as vegetation type. As such, we investigated habitat requirements in the context of predators, microhabitat structure and food for *E. s. badia*. We strongly advocate the structural framework proposed by Armstrong and Seddon [36] in application to a mitigation translocation (Figure 5), which has broad implications for the protection of threatened species. As mitigation translocations continue to be used as a compensatory measure for the ‘rescue’ of threatened fauna at sites marked for development, this research program makes it clear that a ‘continue as normal’ approach, where the speed and scope of infrastructure development sets the pace for managers to conduct translocations in an ad hoc manner without feasibility analysis [93], is no longer acceptable [33]. A significant investment into the planning, design, implementation and monitoring of translocation events is required for mitigation translocations to be considered an effective management tool [33].

## 4. Looking Forward

The targeted research in this program has helped to identify specific ecological requirements of *E. s. badia* for consideration during translocation: (1) predators to target for management, (2) microhabitat structural requirements of a translocation site, and (3) food species required at or near a release site [27]. While each of these is key to an ecological understanding of the skink, they only help to answer the third question (regarding the habitat conditions) of the ten questions considered critical to maximise translocation success [27,36] (Figure 5). Therefore, while our knowledge of the translocation requirements of *E. s. badia* has increased, continued research into further understanding the ecology and optimal translocation requirements of this skink is important to maximise the likelihood of successful management into the future [27] (Box 1).

Box 1 **Recommendations for additional steps to likely improve future *Egernia stokesii badia* translocation success.**

1. Improve pre- and post-release management, such as trialling:

• Soft-release e.g., [94,95]

• Methods to prevent or quantify ‘fence-pacing’ [96]

• Predator deterrents (e.g., overhead wires; [97,98])

• Stress mitigation techniques that are not always intuitive for reptiles [5,99]

2. Better understand metapopulation dynamics, including:

• Colony home range size (to help understand ecosystem carrying capacity [100,101]

• Dispersal capacity (e.g., using radio tracking; [102,103])

• Genetic composition of colonies (e.g., extracting skink DNA from scats; [104])

3. Follow adaptive management:

• Determine life-history strategy (e.g., reproductive requirements and recruitment-rates; [35])

• Consider climate change (e.g., mechanistic species distribution modelling, [105])

• Look into the potential of a captive breeding program, e.g., [106,107]

• Follow an experimental, scientific approach to trials [6,31,108]

4. Monitoring

• Focused and targeted to address questions identified a priori [108]

•Explicitly test and compare the effectiveness of different management alternatives [6]

• Hypotheses for testing must be incorporated into the translocation planning and budgeting process [34]

### A Broader Perspective

The *E. s. badia* case study provides a broader ecological knowledge base to help improve the likelihood of successful translocations for this subspecies in the future. In many parts of the world, translocation “success” is identified by legislators and policy makers as the release of animals. From the perspective of ecological restoration and biodiversity conservation, however, releasing animals is the starting point, and “success” is regarded as the ongoing survival and persistence of the translocated individuals, and the establishment of self-sustaining populations integrated into the metapopulation matrix of the species as a whole [3,36]. While this is amongst the first research studies that aim to align mitigation translocation research and planning to the high standards expected of conservation translocations [33,36], there are numerous examples of how an improved ecological understanding has enhanced translocation success. By following the high standards of conservation translocation and adhering to a scientific framework to test and understand species-specific requirements, this is likely to help address the prevalent issue of many reptile translocations ending in failure [14,109].

Improved understanding of microhabitat requirements has improved reptile translocation success in the past. For example, survival rates of the threatened Florida sand skink (*Plestiodon reynoldsi*) were linked with increased habitat heterogeneity provided at the translocation site, indicating the importance of understanding the microhabitat requirements of the target species [14]. A lack of understanding of the specific habitat requirements, and habitat-related factors, has also strongly influenced declines post-translocation [40]. For instance, translocated Texas horned lizards (*Phrynosoma cornutum*) were found to largely avoid the habitat modified for their benefit during a translocation trial, indicating the need for more detailed studies [110]. Many reptile species are habitat specialists, e.g., [111,112], meaning that ensuring there are specific structural and microclimate characteristics within the habitat can be critical to the successful establishment and persistence of translocated individuals. Overall, targeted research or experimental trials may be required to determine the microhabitat requirements of the target species and optimise the likelihood of a successful translocation.

Globally, invasive species are a cause of herpetofauna decline post-translocation [40]. Translocation losses or failure can result from translocated individuals subject to predation, e.g., [113,114,115]. Predator exclusion and control has been an important component of numerous reptile translocations, e.g., [115,116,117]. Native predators can also be an issue for translocated reptiles, and investigation into predator–prey dynamics can be important to reduce the likelihood of translocation failure, e.g., [118]. Knowledge of key native predators can help to inform translocation location and timing. For example, it can be important to avoid areas of highest predator density, select translocation sites where there is the highest availability of refuge from predators (or create artificial refuges), or release individuals outside of the peak predator breeding season [118,119]. Overall, knowledge of predator–prey dynamics, both with native and non-native predators, improves translocation strategies for reptiles.

An understanding of dietary requirements is important for translocation planning and monitoring. For example, an understanding of diet was important for assessing the post-release dietary similarity of translocated versus resident crocodilians as a measure of translocation success in the critically endangered Philippine crocodile (*Crocodylus mindorensis* [120]). This was a useful alternative to fecundity as an assessment measure due to the long lifespan of the species that would require decades of monitoring data using conventional analysis of reproductive success [120]. An understanding of dietary requirements is also proposed to be important for the selection of translocation sites for tuatara (*Sphenodon punctatus*) in the context of climate change, as they do not digest food at temperatures below 12 °C [121]. Diet specificity in the Fijian crested iguana (*Brachylophus vitiensis*) is also highlighted as a limiting factor for the future selection of translocation sites to improve the likelihood of translocation success [122]. Overall, a more comprehensive understanding of habitat-related factors, including diet, predator–prey dynamics and microhabitat requirements, increases the knowledgebase required to optimise translocation success for reptile species around the world.

## 5. Conclusions

This research program was part of the first efforts into aligning mitigation translocation planning and research with the high standards associated with conservation translocations [36]. Without an adequate investment of time and funding towards a clear understanding of species requirements, translocation for the protection of reptiles runs the risk of being an inadequate use of conservation funding and time [27]. This is particularly relevant to reptile translocations in Australia, which is a global hotspot for reptile richness [19,20], yet suffers from a chronic knowledge gap surrounding the reptile conservation status and ecological requirements [20,27]. However, Australia is not the only region globally where such challenges apply, and similar limitations are likely to emerge in places such as South America and parts of Africa, particularly as the understanding of reptile biodiversity in these regions expands [123,124,125,126].

The use of *E. s. badia* as an Australian case study has provided a clear strategic framework for the targeted research of meaningful ecological factors that influence on-ground translocation decision-making [27]. For example, while introduced predators were confirmed to pose a potential threat to both established and translocated populations, we found that even native predators, in this case native corvids, can become an issue when populations are artificially augmented through the provision of anthropogenic food sources, such as landfill sites [27]. The microhabitat structure of potential reintroduction locations was demonstrated to vary in importance, but in very subtle ways between inhabited and uninhabited sites. The novel application of terrestrial LiDAR was confirmed as an effective tool to quantify structural microhabitat requirements and is a method with extensive applications for the assessment of complex microhabitat types vital to the persistence of other threatened species [27]. Lastly, the complementary use of visual identification and DNA metabarcoding was useful for the identification of a largely herbivorous diet by *E. s. badia*, supplemented by the consumption of invertebrates [27]. The application of this approach to refine the understanding of other species’ dietary requirements has clear facility in guiding translocation planning. The research program provided detailed ecological information that substantially increased the ecological knowledgebase for this endangered subspecies and identified further knowledge gaps that require ongoing research attention. Similar approaches applied to other threatened reptile species are likely to fundamentally increase the capacity for effective management, and the likelihood of successful translocations in the future.

## Data Availability

The datasets generated during and/or analysed during the current study are available from the corresponding author on reasonable request.
